# A Qualitative Study on Danish Student Pharmacists’ Attitudes Towards and Experience of Communication Skills Training

**DOI:** 10.3390/pharmacy7020048

**Published:** 2019-05-21

**Authors:** Neeltje P. Duijm, Karin Svensberg, Casper Larsen, Sofia Kälvemark Sporrong

**Affiliations:** 1 Social and Clinical Pharmacy Group, Department of Pharmacy, Faculty of Health and Medical Sciences, University of Copenhagen, 2200 Copenhagen, Denmark; ksb560@alumni.ku.dk (C.L.); sofia.sporrong@sund.ku.dk (S.K.S.); 2 Section for Pharmaceutics and Social Pharmacy, PharmaSafe Research Group, Department of Pharmacy, University of Oslo, NO-0316 Oslo, Norway; karin.svensberg@gmail.com

**Keywords:** student pharmacists, interpersonal communication skills training, pharmacy education, attitudes, experiences

## Abstract

As the pharmacy profession evolves, good communication skills are vital for securing the safer and more rational use of medicines. Currently there is a lack of qualitative studies researching European student pharmacists’ and their experience with communication skills training (CST). This qualitative study aimed to fill this gap by exploring Danish student pharmacists’ attitudes towards, and experiences of, CST. Focus group interviews were conducted with a heterogeneous sample of Danish student pharmacists in 2016. Interviews were audio recorded, transcribed verbatim and analyzed inductively. Fifteen students participated in three focus groups. Five categories identified as key aspects were: professional communication vs. normal conversation, motivation to engage in training, how to learn communication skills, experience with CST and universities’ role in teaching communication skills. In conclusion, there were both positive and negative attitudes towards CST among the participants. However, they had little experience with CST. Bloom’s taxonomy of the affective domain and Kolb’s experiential learning model appear to be useful in understanding students’ attitudes towards CST. Pharmacy educators can use this study to structure and improve their CST curricula by knowing what influences students’ attitudes towards CST.

## 1. Introduction

The pharmacy profession worldwide has for decades been in a transitional phase towards pharmacists practicing pharmaceutical care [1,2,3]. Both the World Health Organization and the International Pharmaceutical Federation have endorsed this change in practice [4,5,6,7]. Good communication skills are vital when evolving the profession with the aim of the safer and more rational use of medicines, especially when improving patients’ adherence and knowledge about their medicines [6,8,9]. In Denmark, there is also an increased focus from the Danish government on pharmacists’ ability to communicate with patients about their use of medicines [10]. The complex and dynamic interplay between practice, regulation and education indicates that educational components are bound to change as well, for example, by focusing more on students’ communication skills [7,11]. Still, in most European countries, pharmaceutical manufacturing is the main focus in pharmacy curricula compared to the U.S., Australia, Canada and the UK [12,13,14].

Experience with medical students has continuously shown that experiential learning, including feedback, gives a greater improvement in communication skills compared to traditional didactic lectures [15,16]. In a review by Wallman et al. it was found that communication skills training (CST) in pharmacy schools differs a lot between countries and that many articles published focus on the simulated or standardized patient interaction for CST [17]. The researchers found that regarding interpersonal communication skills, teaching was highly focused on practical matters such as learning interview techniques, consulting patients, participation in seminars, simulations, or role-play with peers. However, their results were dominated by American studies [17]. As mentioned, pharmacy schools in the U.S., Australia and Canada have a stronger focus on CST, and in comparison, student pharmacists in the Nordic countries appear to receive little experiential CST [18,19].

Students’ attitudes towards communication skills impact their learning and future counseling behavior [20]. A study on undergraduate medical students’ attitudes towards communication skills learning found that the students held a negative attitude towards subjects containing, what they believed to be “soft science” (e.g., communication skills) compared to “hard science” (e.g., chemistry) [21]. A quantitative American study that measured student pharmacists’ satisfaction with communication teaching methods found that students are more satisfied with nontraditional and non-didactic CST [22]. This is similar to the findings in the two quantitative Nordic studies, which concluded that student pharmacists in the Nordic countries were not satisfied with the patient directed CST they had received and that students had moderately positive attitudes towards CST [23,24]. Factors associated with positive attitudes towards learning communication skills were a newer pharmacy program, being female, acknowledging a need for improving one’s communication skills and believing one’s communication skills are not the result of personality [24]. Currently the vast majority of research on communication skills and student pharmacists explores their attitudes by applying quantitative research methods [22,24,25,26]. Other studies have focused on the specific teaching methods that can be used to structure CST in pharmacy education; such as blended learning, simulated patients and scaffolding [27,28,29]. There is a lack of qualitative studies focusing on student pharmacists’ attitudes and experiences towards CST. Exploring these aspects within a Nordic country could provide more in-depth knowledge on student preferences for CST and reveal other focus areas for educators to consider when structuring and improving CST. Therefore, this qualitative study aimed to extend this knowledge by exploring Danish student pharmacists’ attitudes towards, and experiences of, interpersonal CST. To further extend the knowledge and understand the Danish CST case in an international perspective, the discussion is based on Kolb’s experiential learning model and Bloom’s taxonomy of the affective domain [20,30].

## 2. Materials and Methods

### 2.1. Setting

In Denmark, there are two universities educating pharmacists during a five-year program (three years bachelor followed by a two-year master). The University of Copenhagen admits approximately 240 students per year, and the University of Southern Denmark admits approximately 90 students per year [31,32]. This study was carried out at the University of Copenhagen. In 2016 the University of Copenhagen offered student pharmacists interpersonal CST at two master courses; the compulsory Pharmacy Internship course (30 ECTS) and the elective course Information and Communication about Medicines (7,5 ECTS) [33,34]. One of the objectives of the Pharmacy Internship course was to give students training and skills for dispensing medicine and communicating with patients and other professionals. The course lasted six months. During the internship, students returned to the university and participated in two theory modules. Module 1 lasted five days, and module 2 lasted three days. Each day was dedicated to a topic such as economics, drug dispensing, medication reviews and communication. The students therefore received approximately one day of didactic lectures and classroom teaching in communication. The students could then apply this theoretical learning in their internship [33]. The elective Information and Communication about Medicines course focused on rhetoric, verbal and non-verbal communication about medicines and the uncovering of information needed for different target groups. This course mainly included lectures but also used classroom teaching and admitted around 30 students per year [34].

### 2.2. Design and Interview Guide

A qualitative study was conducted using focus groups. This method was chosen due to its usefulness for exploring not only what participants think, but also how and why they think this [35,36]. A semi-structured interview guide was developed based on the research from Svensberg et al. and discussions in the research team [23]. Consent forms, also containing socio-demographic background questions (gender, age, which year they started their pharmacy education, if other education was completed, and if the elective communication course had been followed) were developed. The interview guide started with a question about the value of communication skills for professional life, followed by specific questions about CST: (1) is the training at the university through mandatory or elective courses, (2) what is the impact of training for improving skills and (3) what role should the university have in developing students’ communication skills? In addition, probing questions were used for clarification of answers and to get more in-depth data.

### 2.3. Ethical Considerations

Ethical approval was not needed according to Danish regulations. Ethical considerations were however met. All participants signed informed consent documents. Data was processed and stored confidentially in accordance with the Danish Data Protection Agency. Approval on processing of personal data was granted by the University of Copenhagen.

### 2.4. Sample Selection and Reqruitment

Student pharmacists at the University of Copenhagen were invited to participate. Inclusion criteria were that they should be pharmacy master students in their last semester or have finished their education within the last year. This was to ensure that they had finished all compulsory communication teaching and could reflect on their education as a whole. Heterogeneity of the sample was pursued for in regard to gender, master thesis subjects, age and if they had or had not followed the elective communication course.

A combination of convenience and snowball sampling was used [37]. A total of 175 e-mail addresses were obtained from a closed group for the Pharmacy Internship course of 2015 and through contacting various departments at the faculty. Invitation letters with information about the study were sent out to the e-mail addresses and through social media, such as Facebook groups for student pharmacists, and handed out at student social events.

### 2.5. Data Collection and Analysis

All focus group interviews were conducted at locations belonging to the University of Copenhagen. The first author moderated all interviews. To aid the moderator an observer (third author) was also present, taking notes and asking additional questions. After completion of each interview, topics and participant dynamics were discussed and elaborated by the moderator and observer [38]. Focus group interviews were conducted in the period from April to June 2016.

All focus groups were conducted in Danish. They were audio recorded and transcribed verbatim. Transcriptions were validated by repeated listening. The transcripts were analyzed by reading them through, marking relevant passages and providing them with a code. Coding, included as part of the following content analysis, was performed inductively by the first author [39]. No software was used for transcription or analysis. It was a dynamic process between the different potential categories. This analysis was discussed between all authors in several consensus discussions. The observer (third author) checked that the results were consistent with the data, and that all relevant data were included [40]. In this study, a communication theory or model is defined as an explanation of information flow between two parties: how is information sent and received, and what factors might influence this.

### 2.6. Interpretation of the Results

To complement the interpretation of the results, two theoretical models were used: Bloom’s taxonomy of the affective domain and Kolb’s experiential learning model. The two models are presented in the following.

Bloom’s taxonomy of the affective domain represents objects concerning attitudes and feelings, and explains the concept of internalization [20]. The affective domain concerns the learning of different values and perspectives, and how these gradually become a part of one’s consciousness to a point where they become an integrated part. Using this perspective for CST is apparent, since communication theories and models are supposed to become an integrated part of students’ professional communication skills. One category from the affective domain is “Responding” towards a phenomenon. This category consists of the levels “Acquiescence”, “Willingness to Respond” and “Satisfaction in Response”. Within the first level, “Acquiescence”, a student can be passive and unwilling to learn. This is contrary to the next level, “Willingness to Respond”, which entails a voluntary activity related to the learning. Hence, when a student voluntarily engages in the learning of new values the student moves from level one to level two. The third level, “Satisfaction in Response”, includes an additional element to the previous subcategory and indicates the student’s positive feelings of satisfaction or enjoyment that follows the voluntary response or consent. This step is an important step in learning since it has self-reinforcing qualities [20].

The experiential learning model developed by Kolb is based on attaining knowledge by reflection on concrete experiences [30]. Firstly, experience is the basis for reflection. Reflections are then concentrated into abstract concepts, which define a new basis for meaning of action (knowledge). This new knowledge then serves as a guideline for new experiences. New knowledge will be tested and reflected as new experiences create new knowledge and alter knowledge already attained [30].

## 3. Results

In total, three focus group interviews were conducted with five, four and six participants in each group, resulting in 15 participants. The focus group interviews lasted on average 104 min. The participants were recruited through e-mail (n = 8), social student events (n = 5) and social media (n = 2). Student characteristics are shown in Table 1.

One important result was the outcome of the interactions in the focus groups. The participants progressed in their understanding of communication, CST and its importance during the focus group sessions. At the beginning of the interviews, it seemed hard for all participants to grasp what CST was or could be about. In all three groups, the discussions at the beginning of the interviews were about how communication was taught with a general academic approach and that it was good to have some abstract tools and techniques for how to interact with patients/customers. However, while the focus group interviews evolved, an agreement arose about the need for practical training of communication skills. At the end, the different groups, independent of each other, all shared the viewpoint that communication skills were also a practical subject, which could be learned through experience.

In total, five categories were identified. All participants appeared to agree that some sort of communication skills was important for all pharmacists, whether working in a hospital, a pharmacy or in the pharmaceutical industry. While speaking about the ability to learn or improve communication skills, some important aspects emerged. Firstly, the participants did not seem to differentiate between a normal conversation and professional communication. Secondly, they mentioned their own willingness, or lack of, to actually take part in CST. Thirdly, they believed that practical training, and not theoretical teaching, was the only way to learn or improve communication skills. These aspects are further elaborated in the categories “Professional Communication vs. Normal Conversation”, “Motivation to Engage in Training” and “How to Learn Communication Skills”. Further, the categories “Experience with CST” and “The Universities’ Role in Teaching Communication Skills” put a perspective on the participants’ experiences with CST and how they thought it should be taught. Translations of quotes from the pharmacist students are used either to emphasize a particular statement from an individual or as general illustrations of a category.

### 3.1. Professional Communication vs. Normal Conversation

In the focus group interviews, most participants stated that they reacted intuitively when counseling a customer, and that communicating with customers at the pharmacy was about having a normal polite conversation. Only once, it was mentioned that a pharmacist should have a “somewhat more professional role” when communicating with customers.

*“Yes, but that is more or less just a conversation where you adapt a bit of the professional role.”*

*(F1B)*

When standing at the counter interacting with a customer, participants found it difficult to apply specific communication theories or models. As an example, one participant explained that no matter what theoretical approach was used; sometimes customers just did not understand what the pharmacist student tried to communicate to them. The participant therefore explained the importance of doing a need assessment, rather than applying theory, when dealing with customers who had trouble understanding the information given.

*“We have had some models, but when you get out there it has been my experience that you are just a normal decent human being”*

*(F1A)*

*“You have to do a need assessment instead of standing there with some random communication models, which you can’t use for anything anyway.”*

*(F1B)*

### 3.2. Motivation to Engage in Training

In one focus group interview it was said that everything can be learned, it just takes willingness and engagement before it is acquired.

*“You can learn how to improve it [communication skills], but I also think that somehow, I don’t know, some willingness in relation to learning and so on.”*

*(F3R)*

*“It’s about training; it’s about being committed and practice it.”*

*(F2F)*

A general attitude across the focus groups was that the subject communication skills was seen as a “fluffy” subject rather than an academic subject. Participants expressed that this might be due to its closer relation to human sciences than natural sciences. The lack of hard facts was frustrating for some and therefore there were difficulties in taking the training seriously. Additionally, it was discussed that most newly graduated Danish pharmacists work in the pharmaceutical industry rather than in a community pharmacy or hospital setting. Participants therefore did not believe that student pharmacists, during their education, felt the need to learn good communication skills. In one focus group, a participant realized that the reason why she did not want to spend time on CST was because she felt she already had the communication techniques she needed to be a good pharmacist.

*“I am really not interested in learning it, that about communicating. /…/ Because I couldn’t see what we need it for.”*

*(F1B)*

### 3.3. How to Learn Communication Skills

When speaking of the possibility to learn communication skills, a general opinion was that it could be learned, or at least improved, with a lot of exercise and training. However, in all interviews, participants expressed that personality and empathy are important aspects regarding the ability to become a good communicator. It was also expressed that some individuals might have a certain flair for communication while others would not.

*“I think there are some who are more naturally talented than others, but I think everyone can acquire the right tools to be good at it [communication].”*

*(F2I)*

A general dilemma brought up in all focus groups was that while communication skills probably could be learned, the participants did not think there was a good way to learn it.

*“I think a part of the problem is that, there is no good way to train it really.”*

*(F1A)*

*“This [the CST experienced at the university] is kind of my only frame of reference, I am not really sure about how it could be done in a different way, I haven’t tried anything else.”*

*(F3L)*

*“I think that is why we are a little vague about it [learning communication] now, because we ourselves are reflecting upon it [how to learn] as we speak. I believe.”*

*(F2I)*

To this, a few participants expressed that if they could learn how to behave in the laboratory, and how to titrate, they could also learn how to communicate properly. In one focus group interview, it was discussed that communication was thought to be a more practical, rather than theoretical, subject. The participants thought that communication models or theories could be used to visualize and reflect upon situations but that they often were not fully applicable in reality. After discussion of communication skills and training, all focus groups came to the conclusion that standing at the pharmacy was the only way to really learn communication skills, by getting experience and learning from mistakes. In essence “learning by doing” at the pharmacy was the only way of properly learning how to communicate with customers according to the participants.

*“If you compare it to the laboratory work we’ve had, right, then we also had some theory at the lectures, and then we tried it in the lab; that does not mean we are Danish champions at titrating right? But it is kind of the same thing here, right. We get some theories, then we try it in practice afterwards at the pharmacy, and then later you need to specialize in communication if that is what you want.”*

*(F1D)*

*“I think a lot of it was very theoretical. In general, I think that communication is practical so I think it gets like the theory of communication, I think it becomes a bit boring, but also difficult and hard to apply in practice.”*

*(F3L)*

One group discussed the need to be pushed out of one’s comfort zone to increase the interest of learning something new. One example was by changing the setting for the CST. A change of scene, outside of campus, was thought to make student pharmacists more open to learn a “soft” science subject, which they believed communication skills to be. Another possible tool for teaching the discussed was for students to videotape themselves during patient counseling (with real patients, students or actors) and use this for improving. When speaking of role play, participants envisioned many possible obstacles and some considered it to be too far out of their comfort zone.

*“Like it is important to find the balance between receiving some tools that you can work with and try it in practice and get some personal feedback. Because that is what you really learn from. You don’t learn something from [looking at] someone standing and doing stuff that you are well aware that you shouldn’t do, but as soon as you get it told yourself [i.e., personal feedback], then you learn something.”*

*(F3M)*

### 3.4. Experience with CST

There were doubts among the participants about if they had really received CST at the university at all. After discussions, all participants identified the pharmacy internship as the main course for CST, specifically one day during the theory modules. According to the participants, the CST they received that day mainly consisted of lectures on theoretical aspects and communication models given by a teacher who had an education both in communication skills and in pharmacy. Some participants expressed positive attitudes towards the lectures. They appreciated the focus on the many different aspects relating to customers, such as customers’ view of the world and that everyone has different struggles in life. One participant said that these lectures about communication theory should not be underestimated, since they probably, unconsciously, picked up something of value for their internship.

*“Definitely, when I use them [the theories] in practice then I thought that it worked. Then there are at least some things you become aware of too, then you probably get some bad habits and so on, we all do, but at least it is something one thinks about and that you take with you, I think.”*

*(F3O)*

A more general tendency was a negative attitude towards the communication teaching received. For example, the content was criticized for being too simple, on a low academic level and participants not being activated in the communication teaching, as they were just listening to lectures. Most participants did not believe they proactively used the theoretical information given in the lectures when interacting with customers at the pharmacy.

*“I didn’t use them [the theories] I would say”. “I don’t think I did either”. “I don’t think I used them [the theories] consciously.”*

*(F3Q, P, M)*

*“I think, or at least as I recall it, I remember it [CST] to be very pedagogical. That it wasn’t like a communication tool, but more like a ‘this is how you speak nice and friendly to the customers’ or like. I don’t remember that I got a tool out of it.”*

*(F2I)*

The participants agreed that the improvement of their communication skills could not be attributed to lectures but rather to the experiences from the pharmacy counter during the pharmacy internship. They appreciated the feedback from their pharmacy supervisors and believed that it helped them develop their skills. Observing other staff members, and getting feedback and information from them, was also highly valued.

*“I don’t necessary think it [improvement of communication] has something to do with it [the theory module at the university during internship]. Not in my case at least. I just think it was getting more and more experience.”*

*(F2I)*

### 3.5. Universities’ Role in Teaching Communications Skills

There were two dominant contradicting viewpoints on the role of universities. One viewpoint was that the university mainly should give an academic and theoretical education. Practical matters, such as behavior, and thereby communication skills, should be learned either before or after the university education. Hereby student pharmacists could choose if they wanted to learn communication, depending on their future work in a pharmacy, clinical setting or pharmaceutical industry.

*“I look at the university a bit like, it is a theoretically based education right. So well, all of the practical stuff, that might be something you learn besides from it or afterwards”*

*(F1D)*

The other viewpoint was that the university should prepare students for all future job opportunities upon graduation. The university therefore has a great responsibility teaching every aspect of the pharmacy profession, whether this is working in the laboratory or communicating with customers.

*“I think they [the university] have a small role, well they are supposed to, I understand that we should be more independent, but they should still prepare us for when we finish. It is an education we are taking, so well, they should prepare us for handling a job later on. So I definitely think that the university should play a role in it.”*

*(F2G)*

Due to the viewpoint that the university was mainly theoretical and reflection based, and not practical, there were doubts about the responsibility for the university to teach communication skills. However, it was brought up that student pharmacists already learn many practical things whilst standing in the laboratory, applying learned theory to work with chemicals.

*“The practical stuff like laboratory-work, that you can’t [learn without practical training] either. You can’t just sit and read McMurry [chemistry curriculum] three times in a row and then you are a champion in the laboratory.”*

*(F1D)*

The participants said that it was not possible to learn how to act in all situations at the university, since you could not foresee what sort of situations would happen out in the working field. But the university could provide some good tools in preparing and giving students time and the possibility to reflect, evaluate and receive feedback, and therefore to improve.

## 4. Discussion

This qualitative study aimed to explore Danish student pharmacists’ attitudes towards, and experiences of CST. Overall, the participants expressed that communication skills were important. However, they lacked a clear understanding of CST, and what communication skills are required in professional pharmacy counseling sessions. Participants were unsatisfied with the level, amount and content of CST received. At the same time, there were opposing beliefs towards whether the university should provide CST to student pharmacists or not. It seemed that participants experienced a lack of alignment between theoretical teaching and practical learning. There also appeared to be lack of reflection on communication skills among the participants (e.g., as how it could be used as a tool to improve patient outcomes).

**Figure 1:**
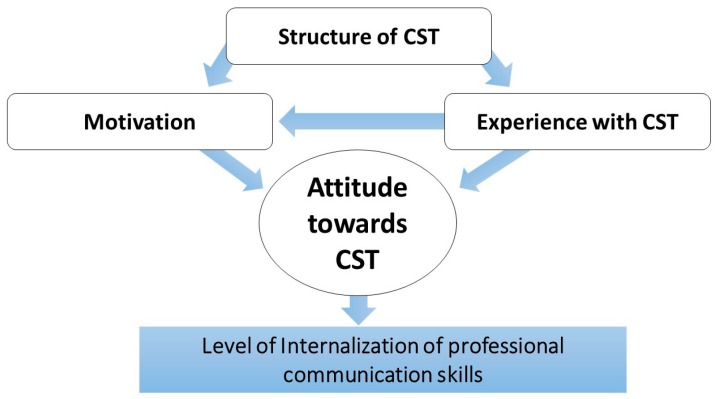
The relationship between students’ motivation, experience and attitude towards CST (communication skills training).

This study found the relationship between students’ motivation, experience and attitude towards CST (Figure 1). If participants view communication skills as being “fluffy” and unnecessary for pharmacists working within natural science, their motivation to engage in CST decreases as well as their positive attitude. A lack of CST experience during their education also influences student pharmacists’ ability to reflect upon the need for good communication skills, thereby influencing their motivation and attitude. These concepts are further discussed in relation to Bloom’s taxonomy of the affective domain and Kolb’s experiential learning model to put a theoretical perspective on the Danish CST case [20,30].

### 4.1. Bloom’s Taxonomy of the Affective Domain and Internalization

By interpretation of the study results, participants were between level one (Acquiescence) and two (Willingness to Respond) in Bloom’s taxonomy. It seemed participants understood that they have to play an active part to develop their communication skills. However, even though they did see the necessity of communication skills, some did not want to engage in such training. Reasons for a lack in willingness were influenced by the perception that communication skills only partly can be learned. Even though it was viewed as an important skill, the need to learn was not present. These factors were also found influencing Nordic student pharmacists’ attitudes towards learning communication skills [24] (i.e., if students saw a need to learn communication skills, and believed that it could be learned, they were more positive towards CST). Not feeling the need to learn communication skills could be due to the student pharmacists’ dissatisfaction after participating in CST sessions, influencing the perceived relevance of CST.

The view of CST being on a low academic level and common sense is similar to the findings by Rees et al. [21]. The researchers found that medical students had trouble taking communication skills teaching seriously, as they did not see communication skills as a pure science subject. The similarity between pharmacist and medical students’ attitudes towards communication skills is interesting. It could indicate that students’ in natural sciences share a negative preconception of some areas of human sciences. This influences their ability to reach level two, “Willingness to Respond”, in Bloom’s taxonomy of the affective domain. When developing curricula, this negative preconception must be taken into account as well as how the CST is introduced. Good experiences with CST could improve the students climbing up the ladder of the taxonomy, internalizing professional communication skills.

### 4.2. Kolb’s Experiential Learning Model and CST

During the interviews, participants stressed the usefulness of individualized feedback in order to learn and improve their skills. Participants found it difficult to apply learned communication theories and models to practice. Following Kolb’s experiential learning model, experiential sessions with feedback would allow students to reflect actively about their experiences and train them to communicate in alignment with the theoretical knowledge provided by the university.

Within medical education, as well as pharmacist education, receiving and providing feedback on communication skills during experiential teaching methods, both with peers or teachers, has been shown to be effective and is widely incorporated [15,16,41,42,43,44,45]. Planas and Er [29] conclude that CST with self-directed learning, feedback and scaffolding strategies are “effective in helping students recognize their communication strengths and areas in need of improvement as well as implement and evaluate strategies to improve their communication skills” (p. 10). Furthermore, Svensberg et al. found an association between Nordic student pharmacists following a larger and more varied CST program at the university and more positive attitudes towards this training compared to students mainly receiving didactic and less teaching [23]. Participants in this current study explained the need to be pushed out of their comfort zone to learn. This could also explain why a varied CST program, entailing experiential methods and feedback are helpful when teaching communication skills.

### 4.3. Practical Considerations

In the present study, despite having trouble defining CST, some participants clearly stated that they did not feel the university prepared them for the pharmacy internship at a community pharmacy. Most did not consider one day of specific communication teaching to be sufficient preparation for counseling situations. An opposing view was that the university should not prepare students for the practicalities in pharmacy practice, as the university should be theoretical and not practical. This view was surprising, since their bachelor courses (e.g., chemistry and manufacturing), included practical laboratory components. The rationale behind this statement might be that there is no step-to-step manual for how to communicate in every given situation. Participants stated that they are used to following specific procedures with right or wrong steps. Participating in a subject which is dependent on your ability to reflect and structure arguments for your case, could pose a challenge. However, there are practical guidelines and methods developed providing hands on approaches for pharmacists in consultations, such as the motivational interviewing technique and the Calgary–Cambridge guide, which has been proven effective [46,47]. More focus on practical guides could improve pharmacy students’ motivation during CST courses, which then might improve their attitudes (Figure 1). Studies have proven that communication skills can be learned and developed in educational settings [8,16,41]. Aspegren’s review of teaching and learning communication skills in medicine establishes that experiential teaching methods succeed the use of instructional methods regarding effectiveness [16].

It is important to consider how these experiential learning methods and techniques are used. Harlak et al. investigated the attitudes of medical students towards CST before and after participation in a CST course [25]. They found that positive attitudes had decreased over the course and implied that the curriculum needed modification. Their results indicate that if not structured in a relevant way to students, they might end up with more negative attitudes towards CST than before they entered the course. Participants in the present study were very keen on “learning by doing” at the pharmacy. Role play, is a method to “learn by doing” since the students will get familiar with constructing conversations in a safe setting. It could even be more beneficial with CST in a safe environment, since it will provide the opportunity for more structured feedback, and no harm will come if mistakes are made. However, the structure and introduction to such a learning method, needs to be carefully considered to maintain a positive attitude among students. Especially since participants in this study were skeptical towards the idea of role play.

### 4.4. Practical Implications and Main Challenge

This study identifies one main challenge for educators: to structure the communication experiences in a way that positively influences and reinforces students’ motivation and attitude towards the training, so they can internalize professional communication skills (Figure 1). The main challenge can be divided into three aspects for educators to consider:

- Motivation: Getting all students to understand the importance of professional communication for a pharmacist, that this is learnable, and not something you are born with.
- Structuring CST: Structuring the training in such a manner that every student understands the necessity of it. Communicating this explicitly to the students so they feel they can use the feedback and knowledge constructively during their interactions with customers or other professionals.
- Experience: The Danish students have (almost) no experience of CST that they can relate to when discussing this subject. Hence, courses should be organized so that the students have some experience prior to theoretical teaching.

That Danish student pharmacists have relatively little experience of CST could explain that they are rather skeptical to new teaching methods. However, they themselves identified a need to get pushed out of their comfort zone to learn. Experiential teaching methods can be useful in this process but communicating the necessity of the training to student pharmacists should not be underestimated when improving teaching.

For further research, it would be interesting to investigate the motivational aspect of CST and if factors found in this study influence motivation and attitudes towards CST in other countries. It could be interesting to compare motivation levels between countries and if this can be explained by differences in curricula or if there are other factors associated.

## 5. Limitations

This study has some limitations. Participation was based on convenience sampling and only from one university, out of two in Denmark, educating pharmacists. It is possible that students taking part in the focus groups are more positive towards the topic compared to non-responders. This could have led to data saturation that was not reached, and that there are aspects not accounted for in this study. However, participants expressed both negative and positive attitudes, which strengthens the quality of the results. Since this study is exploratory the findings are not meant to be generalized, but can hopefully be transferrable to other similar teaching settings.

## 6. Conclusions

There were both positive and negative attitudes towards CST among Danish student pharmacists. All participants thought communication skills to be important for pharmacists, however, there was no real differentiation between a normal conversation and professional communication. Furthermore, practical training was believed to improve communication skills rather than theoretical teaching, but the participants also expressed a lack in motivation to participate in practical training sessions. Interesting for educators is that this might be because of a lack of training. It is important for educators to consider how CST is taught to keep students engaged and motivated. More focused, student centered CST at the university would increase students’ knowledge and skills regarding communication and aid their process of reflection on themselves, their actions and the need for professional communication skills. Kolb’s experiential learning model and Bloom’s taxonomy of the affective domain appear to be effective in understanding students’ attitudes towards CST. Using the two theoretical perspectives, educators can deal with anticipated student attitudes and improve students’ motivation to participate in CST. This study can be used as a tool to evolve universities’ local communication curricula.

## Figures and Tables

**Table 1:** Student pharmacist characteristics of the study sample.

Characteristics		Students (n = 15)
Age (years)	Median	24
	Range	23–28
Gender	Women	5
	Men	10
Completed other education	Yes	1
	No	14
Completed elective communication course	Yes	3
	No	12
